# Effects of photochemical riboflavin-mediated crosslinks on the physical properties of collagen constructs and fibrils

**DOI:** 10.1007/s10856-013-5038-7

**Published:** 2013-09-05

**Authors:** Harvey Rich, Marianne Odlyha, Umber Cheema, Vivek Mudera, Laurent Bozec

**Affiliations:** 1 Biomaterials and Tissue Engineering, Eastman Dental Institute, University College London, London, UK; 2 Division of Surgery and Interventional Science, UCL Tissue Repair and Engineering Centre, Institute of Orthopaedics and Musculoskeletal Science, University College London, London, UK; 3 Department of Biological Sciences Birkbeck, Institute of Structural and Molecular Biology, University of London, London, UK

## Abstract

The use of collagen scaffold in tissue engineering is on the rise, as modifications to mechanical properties are becoming more effective in strengthening constructs whilst preserving the natural biocompatibility. The combined technique of plastic compression and cross-linking is known to increase the mechanical strength of the collagen construct. Here, a modified protocol for engineering these collagen constructs is used to bring together a plastic compression method, combined with controlled photochemical crosslinking using riboflavin as a photoinitiator. In order to ascertain the effects of the photochemical crosslinking approach and the impact of the crosslinks created upon the properties of the engineered collagen constructs, the constructs were characterized both at the macroscale and at the fibrillar level. The resulting constructs were found to have a 2.5 fold increase in their Young’s modulus, reaching a value of 650 ± 73 kPa when compared to non-crosslinked control collagen constructs. This value is not yet comparable to that of native tendon, but it proves that combining a crosslinking methodology to collagen tissue engineering may offer a new approach to create stronger, biomimetic constructs. A notable outcome of crosslinking collagen with riboflavin is the collagen’s greater affinity for water; it was demonstrated that riboflavin crosslinked collagen retains water for a longer period of time compared to non-cross-linked control samples. The affinity of the cross-linked collagen to water also resulted in an increase of individual collagen fibrils’ cross-sectional area as function of the crosslinking. These changes in water affinity and fibril morphology induced by the process of crosslinking could indicate that the crosslinked chains created during the photochemical crosslinking process may act as intermolecular hydrophilic nanosprings. These intermolecular nanosprings would be responsible for a change in the fibril morphology to accommodate variable volume of water within the fibril.

## Introduction

Tissue engineering (TE) promises an advanced approach to respond to clinical needs and practices that are currently falling short of expectations. The field combines cell transplantation, biomaterial science, bioengineering techniques and biochemical expertise to deliver both biologic and artificial components for permanent repair, designed specifically for the patient [1]. The ideal properties of a biomimetic scaffold include; good and rapid adherence; ease of handling and application; rapid bio-integration allowing neovascularisation; and, it must provide an environment that supports cell attachment, growth, proliferation and formation of extracellular construct (ECM). The use of collagen constructs as a native scaffold holds tremendous promise as it circumvents the complications associated with synthetic polymers [2–5] and was originally proposed as substrate for cell culture in 1956 by Ehrman and Gey [6]. A collagen construct is defined as an acellular biphasic system consisting of a fibrillar collagen network and fluid components. Recently, the versatility of such collagen constructs for patient’s benefits was reviewed by Abou Neel et al. [7], highlighting the growing range of applications available for collagen constructs in all fields of regenerative medicine. The formation of such constructs is governed by the assembly of the fibrillar collagen structure in a well-defined arrangement, also known as fibrillogenesis. The assembly of collagen molecules occurs spontaneously at neutral pH and physiologic temperature [8, 9]. The self-assembly is therefore a thermodynamically driven aggregation of collagen molecules once the neutralisation of the collagen solution is achieved. In vitro formed fibrils have similar morphologies and characteristic “D-banding” periodicity to those produced in vivo. The fibrils may vary in length and diameter according to the growth conditions and have the ability to form three dimensional supramolecular assemblies (constructs). Assembly methods used to produce fibrillar collagen constructs have been widely investigated using physical and chemical methods such as; nano-manipulation with Atomic Force Microscopy [10]; hydrodynamic flow and microfluidics [11–13]; magnetic field alignment [14]; dip-pen lithography [15] and; chemical nanopatterning [16]. Although current macro- and micro-fabricated approaches have had some degree of success in producing functional collagen constructs, they fail to recreate the natural nanostructures that cells interact with [17]. The extra-cellular matrix (ECM) is responsible for the enhancement of cell proliferation, differentiation and cell-to-cell interaction. Thus, it is an essential requirement for in vitro fibrillar collagen constructs to mimic, both morphologically and chemically, the properties of natural ECM. To date, one of the most challenging properties for the engineered collagen construct to attain is to have the same mechanical properties as their target tissues. This is particularly important to tolerate natural stresses and restore function to a damaged tissue. Self-assembled collagen constructs are mechanically weak due to their fragile lattice structure when compared to natural collagen in vivo. They are also vulnerable to chemical and enzymatic attacks which they would encounter in implantation. Their high hydrophilicity may also cause rapid swelling on hydration, altering their mechanical properties and adversely affecting their process ability [18]. On addition of cells, collagen constructs are subject to cellular matrix remodelling and thus undergo significant contraction as a result. A study by Ibusuki et al. [19] suggested that if constructs could be reinforced to prevent contraction in spontaneous gelation after the addition of cells, cellular mixes in solubilised collagen could be delivered via minimally invasive techniques to fill defects [19]. A promising approach was developed by Brown et al. [20]. In their research, they developed a cell-independent protocol to optimize the mechanical properties of the collagen constructs (termed scaffolds in the original research) by means of plastic compression (PC). Plastic deformation is a permanent change in shape that is non-recoverable once the stress applied in deformation is removed. Excess fluid in the collagen constructs (hydrogel: a result of casting) is expelled resulting in a denser, mechanically stronger structure [21]. Compared to classic conversion processes (involving expensive cell-based remodelling over a period of weeks), plastic compression is a suitable mechanism for increasing matrix and cell density in minutes. The limitation of this approach resides in the fact that individual collagen fibrils are mechanically weak due to the absence of covalent crosslinks between individual collagen molecules. To date, one of the most widely used approaches for producing collagen constructs with improved mechanical properties is cellular matrix remodelling. The cellular interaction with the collagen construct has been reported to produce a modest increase in strength and stiffness when compared with acellular constructs [22, 23]. Furthermore, the length of time required to observe significant improvement can span over several days or weeks. The overall improvement in mechanical properties is also variable and depends on several factors such as: cell type [23–25] and density [22, 23]; passage number [26]; collagen concentration; and the concentration of serum in the culture medium [25, 27]. Another approach to improve the mechanical properties of the collagen constructs is mechanical conditioning. Pre-loading of collagen constructs with loads (compressive or tensile) induces fibril orientation normal or parallel to the direction of the applied load [28]. The produced fibril orientation creates mechanical anisotropy into the constructs and improved mechanical properties in the direction of induced load. This method produces variable results and depends greatly on the construct structure and the loading regime used [29, 30]. The most common approach to improve the mechanical properties of collagen construct involves using chemical crosslinking reagents [31–33]. One of the disadvantages of this approach is that the chemical reagents involved and the reaction products may have detrimental effects on the biodegradation and biocompatibility of the cross-linked constructs. Photochemical cross-linking is considered to be a rapid and efficient process [34] and has reduced toxicity compared with other methods [35]. Photochemical cross-linking has been previously shown to significantly increase mechanical stability (tensile and compression strength), reduce swelling rate and increase longevity in vivo without disintegration [36]. Here, we used this rapid approach for engineering collagen constructs using riboflavin as a photoinitiator to modulate the mechanical properties of the collagen constructs. Although riboflavin may be a non-toxic photosensitizer [34, 35], we have studied its effect on cell viability. In a previous study, we have shown that cell viability (percentage of live cells) drops from 90 to 79 % for fibroblast cells embedded within riboflavin-crosslinked compressed collagen constructs. Although there is a decrease in cell viability, this compromise is offset by the enhanced mechanical properties of the constructs, making it a viable tissue engineering scaffold [37]. More recently, Mi et al. [38], have presented a study in which they engineered plastically compressed collagen scaffolds modified with riboflavin photochemical cross-linking to support corneal epithelial cells. This study is an example of the need for more mechanically stable constructs to be used as scaffolds in tissue engineering. In our study presented here, we focus on the characterisation of the collagen constructs engineered using this growing riboflavin-based crosslinking approach as well as providing answers towards the impact of the newly formed covalent crosslinks engineered on the physical properties of the collagen fibrils present within the construct itself.

## Materials and methods

### Collagen construct engineering

3 ml of rat-tail single molecule collagen type I solution (2.08 mg/ml protein in 0.6 % acetic acid; First Link Ltd, West Midlands, UK) was homogenously mixed with 0.5 ml 10 × Eagle’s minimum essential medium (MEM; Gibco Chemicals, Invitrogen) and 0.5 ml 0.25 mM riboflavin (aq.) (Sigma Aldrich). For control constructs, 0.5 ml of 1× MEM was added instead of riboflavin to make a 5 ml construct. The solution was neutralised drop-wise, with a needle and syringe, using 5 M sodium hydroxide (NaOH) until the first tinge of cirrus pink was seen. Then 1 M NaOH was subsequently added until the phenol indicator changed from yellow/orange to cirrus pink, indicating a neutralised solution. The solution was transferred to a standard mould (22 × 33 × 10 mm) and placed in a dry incubator at 37 °C and 5 % CO_2_ for 30 min to allow fibrillogenesis to occur [20, 39].

### Photochemical crosslinking

The collagen construct as described above included 0.5 ml 0.25 mM riboflavin (7,8-dimethyl-10-(D-ribo-2,3,4,5-tetrahydroxypentyl)isoalloxazine: C_17_H_20_N_4_O_6_) prior to the neutralisation step and was kept in its mould and placed under a lamp (Enfis UNO Air LED Light Engine D006-015C, Enfis Ltd; set to blue light (465 nm wavelength), 4,680 mW intensity, 252 Lumens, 36 W input power). The lamp was a set distance of 10 cm from lamp aperture to bench surface using a clamp and clamp-stand. Photochemical crosslinking was performed either before or after the plastic compression stage and exposure times were varied to produce sample construct at 2.5, 5, 10 and 30 min. Once prepared, the constructs were kept in phosphate buffered solution (PBS) in an optically opaque container at 4 °C for up to 7 days.

### Plastic compression

As described in literature [20], the constructs were removed from the mould and placed on a fine nylon mesh, beneath which was an absorbent pad of Whatman papers. A second nylon mesh was placed on top the constructs before loading the glass slide and 120 g weight. Compression time was 5 min resulting in expulsion of >95 % water to give a thin collagen sample (50–60 μm). Once compressed, samples were rinsed three times in PBS and stored in PBS. Once prepared, all constructs were kept in PBS in an optically opaque container at 4 °C for up to 7 days before being discarded.

### Dynamic mechanical analysis

Plastically compressed collagen constructs (PC) were rinsed in ultra-high purity (UHP) water to remove PBS. Prior to the DMA analysis, the samples were cut into dogbone shapes using a punch: 30 mm (long) × 22 mm wide at the clamp (18 mm in the centre of the dogbone shape) × 50 μm thick. To ensure sufficient grip of the sample in the clamps, additional 2 mm strip of thin steel meshes were glued (cyanoacrylate super-glue) at both end of the collagen constructs. Dynamic Mechanical Analysis measurements were performed on a DMA-7e (Perkin-Elmer). Mechanical testing was carried out under uniaxial tension using static load at a loading rate of 200 mN/min until failure, with constant sample hydration by application of buffer. [20]

### Attenuated total reflectance—Fourier transform infrared spectroscopy

Plastically compressed collagen construct (PC) were rinsed in UHP water to remove PBS and cut to approximately 5 mm^2^ sections using a scalpel. The samples were positioned centrally on the Golden Gate Single Reflection Diamond ATR attachment (Specac, Graseby, UK) in a Perkin Elmer series 2000 FTIR spectrometer (4 cm^−1^—8 co-additions). The sample were fully hydrated during their mounting on the Golden Gate and left to dry on the diamond window for the duration of the experiment, whilst spectra were recorded at given time intervals. The pressure applied onto the samples by the ATR attachment was kept constant. With this technique, the lower few microns of the sample in contact with the diamond window are analysed.

### Atomic force microscopy

For single molecule imaging, a 40 μl droplet of the 1:1,000 solution of acid soluble collagen was deposited on a mica substrate (mica disks; Agar Scientific, Stansted, UK) that had been freshly cleaved. The solution was left to incubate for 10 min, avoiding droplet evaporation by keeping a water droplet at the side of the sample but not in contact so as to avoid sample dilution. The sample was then rinsed, firstly using PBS and then using UHP water to avoid any salt crystal formation. Finally the sample was dried using a gentle stream of dry N_2_. The imaging experiments were carried out using a Multimode-Nanoscope IV (Bruker, Santa Barbara, CA), equipped with an E scanner and NSC tips-D lever (MikroMasch, Tallinn, Estonia). The samples were imaged in dry conditions in tapping mode, with the minimum amplitude set point selected to avoid either damaging or altering the sample on the surface.

For the topological assessment of the collagen fibril formation, a 40 μl droplet of the collagen solution was sampled immediately after the neutralisation step and deposited on a mica substrate. Images were obtained subsequently following the same protocol as for the single molecules. For imaging individual collagen fibrils issued from the collagen constructs and the collagen construct itself, PC were cut to approximately 5 mm^2^ sections and physisorbed onto a glass slide using a gentle N_2_ flow to ensure that the constructs were immobilised which is a prerequisite for atomic force microscopy measurements. Individual collagen fibrils were imaged at the edge of the physisorbed collagen constructs, whereas bulk collagen construct images were obtained in the central area of the 5 mm^2^ section. To do so, a Nanowizard (JPK, Berlin) atomic force microscope was operated both in contact mode using CSC probes (MikroMasch, Tallinn, Estonia) and in tapping mode using NSC (MikroMasch, Tallinn, Estonia). A typical scan rate of 2 Hz was used for contact mode imaging and 1 Hz for tapping mode imaging, whilst the deflection/amplitude set points were reduced to minimize the contact force. Both deflection/amplitude and topography images were recorded. For imaging individual collagen fibrils in a liquid environment, PBS was used to rehydrate the fibrils after they had been imaged in dry condition using tapping mode.

## Results and discussion

### Fibril assembly in a biomimetic collagen construct

**Fig. 1:**
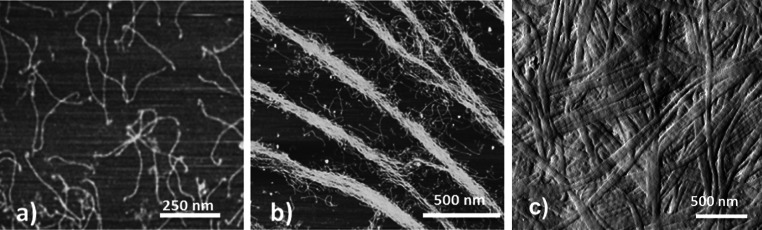
AFM images of **a** acid-soluble single collagen molecule prepared on a mica substrate, **b** collagen molecules packed together side-by-side forming new fibrils, **c** compressed collagen construct displaying fully formed fibrils presenting a D-banding periodicity

The initial stage of the formation of the collagen fibril from a solution of acid soluble single molecules involves fibrillogenesis. The collagen molecule is water soluble in acidic solution (pH 2–3) but insoluble at neutral pH. Acid-solubilised collagen is therefore unable to form fibrils until it is neutralised [40]. Figure 1a shows the topology of collagen molecules prepared on a mica and imaged by atomic force microscopy (ambient conditions) prior to fibril formation. The molecules have a triple helix structure with a typical contour length of 300 nm and a diameter of 1.5 nm as imaged by Mertig et al. [41] and more recently Bozec et al. [42]. Figure 1b shows several three-stranded type I collagen molecules pack together side-by-side to form new fibrils. During the fibrillogenesis, collagen molecules self-assemble into fibrils which in turn form a biphasic system consisting of a fibrillar network and fluid components. The process of fibrillogenesis is driven by the increase in entropy associated with loss of water from the bound monomers [43]. It is the hydrophobic amino acid residues creating weak non-covalent bonds during fibrillogenesis that are responsible for holding the collagen structure together. Thus, the collagen constructs formed in vitro using the protocol described here contains no covalent cross-links between individual molecules making up the collagen fibrils. In vivo, several subsets of intermolecular covalent crosslinks can be formed prior or during fibrillogenesis such as disulphide bridges between sulphurous cysteine residues or lysyl oxidase mediated crosslinks. These crosslinks are stronger that the non-covalent interactions created during the in vitro process and thus confer in vivo (native) collagen fibrils their overall superior mechanical properties.

The newly formed construct can be described as a collagen hydrogel containing 95 % water. This is water content is predominantly responsible for the constructs poor mechanical properties as previously reported [44]. One approach to improve their mechanical properties is to increase the packing density of the construct by means of plastic compression as described by Brown et al. [20]. During the plastic compression, up to 95 % of the interstitial water is expelled from the collagen construct, leading to the formation of a denser sheet of collagen as shown in Fig. 1c. During that process the collagen fibril topology remains unchanged as only interstitial water is removed from the construct. Subsequently, dynamical mechanical analysis (DMA) was performed on the collagen constructs to provide a measure for the bulk Young’s modulus: E_control_ = (249 ± 42 kPa) N = 8. This result is in accordance to previously published data by Cheema et al. [44]. Nevertheless, this value is still low when compared the Young’s modulus of native tissue such as tendons for example, which would have a moduli in the range of 50–150 MPa depending on their role or location in the body. Thus, to further improve the mechanical properties of the collagen constructs, an additional step was introduced to the protocol. This step involves engineering the formation of intermolecular covalent crosslinks by a photochemical crosslinking method directly after the fibrillogenesis. This approach requires using a photoinitiator, Riboflavin (vitamin B2), to release reactive single oxygen in the collagen solution. Accordingly, riboflavin was introduced to the single molecules collagen solution before the fibrillogenesis stage and was activated (λ = 465 nm) either before or after the plastic compression. Upon photochemical activation, oxygen species are released from each of the carboxylic groups present in riboflavin, leading to the generation of a light activated riboflavin and single reactive oxygens in solution (O^2−^). As there are two by-products of the photochemically activation, there are two mechanisms available to interact with the collagen structure. In the direct mechanism (type I), the light activated riboflavin interacts directly with the collagen molecule by hydrogen abstraction of amines to stabilise its own carbon double bonds therefore enabling intermolecular covalent crosslinks to form in the collagen. In the indirect mechanism (type II), single reactive oxygens form hydrogen peroxide (H_2_O_2_) and free radicals (e.g. superoxide anion O^2−^) which in turn oxidise the collagen molecule and therefore enable the formation of indirect covalent crosslinks [45]. It has been proposed that the cross-linking sites in between collagen molecules are nonspecific, and their exact intermolecular location has not been identified [46]. Possible amino acids that are vulnerable to photochemical cross-linking using riboflavin include tyrosine, histidine, cysteine and methionine [18]. The advantage of using riboflavin over other chemicals such as Rose bengal (requiring an argon laser (λ = 514 nm) is that an activation wavelength can be found in the visible region of the spectrum which does not degrade the collagen construct as an UV activation wavelength may. This was tested by performing control AFM imaging both on a crosslinked and a native collagen construct.

### Mechanical response as function of crosslinking

**Fig. 2:**
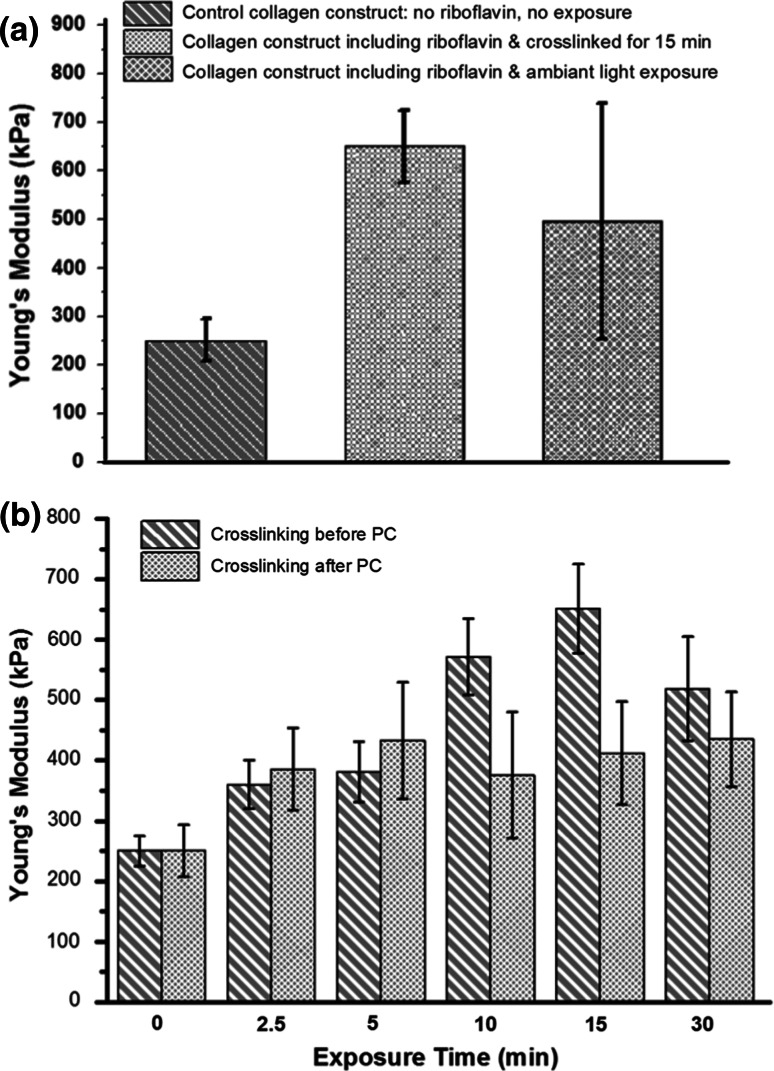
**a** Young’s Modulus values of control, riboflavin crosslinked (15 min exposed to activation lamp) and riboflavin crosslinked (exposed to *white light*) collagen constructs. **b** Young’s Modulus values of collagen scaffold crosslinked either before or after the plastic compression stage. The exposure time is in the *x* axis. *Error bars* represent the standard deviation on the mean value (*P* < 0.05)

Having established the Young’s modulus of the control construct (no riboflavin and no exposure to the activation lamp) using DMA, similar measurements were repeated on constructs crosslinked using riboflavin exposed to either the activation lamp (λ = 465 nm—15 min) or to laboratory environmental white light as presented in Fig. 2a. As hypothesised, the riboflavin crosslinked construct using the activation lamp had a much higher Young’s modulus: E_crosslinked_ = (650 ± 73 kPa) N = 8 when compared the control construct: E_control_ = (249 ± 42 kPa) N = 8. The increase in the Young’s modulus is about 2.5 fold. From these results, it is clear that the addition and activation of the riboflavin in the collagen solution result in the generation of crosslinks which reinforce the collagen constructs and enable it to have a significantly higher value of Young’s modulus. When exposing the collagen constructs containing riboflavin to environmental white light, a significant increase in the Young’s modulus was also recorded: E_whitelight_ = (496 ± 250 kPa). The value of the modulus obtained in this case is statically higher than that of a control collagen construct. The reason for the construct to be crosslinked is due to riboflavin activation by environmental white light containing the 465 nm (blue) wavelengt. The scatter in the values of moduli obtained is due to the fact that photochemical crosslinking activation by environmental light is an uncontrolled a process producing a random crosslinking of the constructs.

The effect of the protocol sequence of plastic compression and photochemical crosslinking was also investigated. Two protocols of crosslinking were devised: photo-activation before the plastic compression stage and photo-activation after. The impact of these two protocols on the Young’s modulus value of the collagen constructs was tested using DMA. As shown in Fig. 2b, the value of the modulus keeps on increasing steadily up to a value of E_crosslinked-beforePC_ = (650 ± 73 kPa) N = 8 for an exposure time of 15 min in the case of the construct crosslinked before plastic compression. No significant increase in the Young’s modulus is recorded when the exposure time is increased beyond 15 min. A possible reason for this is that all single reactive oxygens present in solution (O^2−^) are depleted after 15 min exposure of the collagen constructs to the activation lamp. However, if the compression step occurs before the crosslinking step, then the modulus does not significantly increase regardless of the length of time exposed to the activation lamp. In this case the modulus value is E_crosslinked-afterPC_ = (412 ± 85 kPa) for an exposure time of 15 min. During plastic compression, a considerable increase in packing density is achieved as up to ~95 % fluid can be expelled on a single compression [39]. This process increases the strength and frequency of the inter-fibrillar attractive forces (non-covalent, e.g. electrostatic) as fibrils are brought closer in contact with one another. As a large volume of the solution is expelled during the plastic compression, there is a strong possibility that the photo-initiator is washed out during that step and the remaining volume of photo-initiator present in the construct after compression is fully activated after the first few minutes of light exposure. In contrast, crosslinking prior to the compression ensures that all the initial photo-initiator present in the solution can be used for activation prior to the washout. Taking this into account, it is likely that the modulus of the crosslinked collagen construct could be increased further by altering the initial concentration or volume of riboflavin mixed with collagen construct solution but this may affect the stability of the constructs created as preliminary data suggested (data not shown here).

### Effect of hydration/dehydration on the collagen construct

**Fig. 3:**
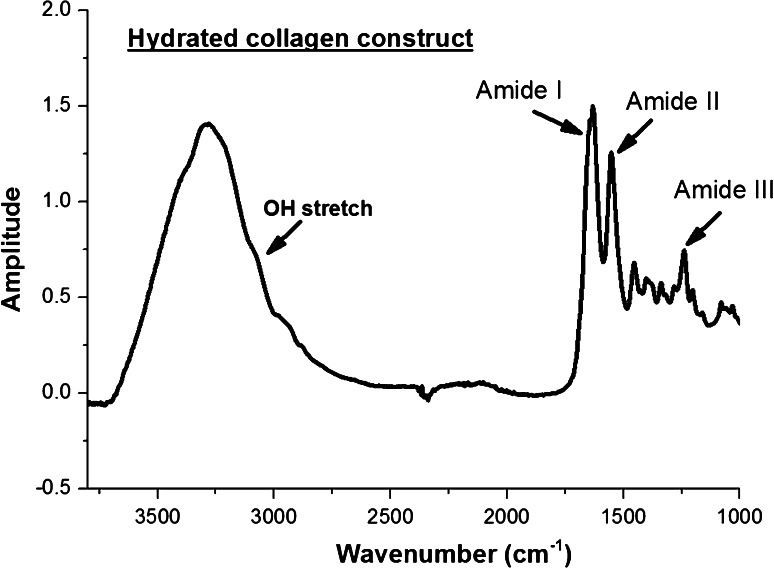
Infrared spectra of a collagen construct fully hydrated. In the case of the hydrated collagen construct, the OH-stretch is a broad band with a centred at 3,200 cm^−1^

A collagen construct is a biphasic system consisting of a fibrillar network and fluid components. Even when compressed, the fibrillar assembly of collagen is highly hydrated with fluids occupying the interstitial spaces. To date, the link between hydrophilicity of collagen and its crosslinks content is not yet clear. In order to assess the impact of using riboflavin as photoinitiator in the collagen construct attenuated total reflectance (ATR) Fourier transform infrared spectroscopy measurements were performed on selected hydrated collagen constructs: control collagen constructs and 30 min riboflavin crosslinked collagen constructs. As anticipated, the fingerprint spectrum of collagen was obtained with characteristic bands as show in Fig. 3: Amide I band: 1,650 cm^−1^ (C=O stretching vibration); Amide II band: 1,550 cm^−1^ (N–H bending vibration and C–N stretching vibration) and Amide III band: 1,230 cm^−1^ (C–N stretching vibration and C–N–H in-plane bonding). It is worth noting that the OH-stretch (broad band with a maximum at 3,200 cm^−1^) is a broad band which overlaps with the amide A band and is used as indicator of the presence of water in the collagen constructs.

**Fig. 4:**
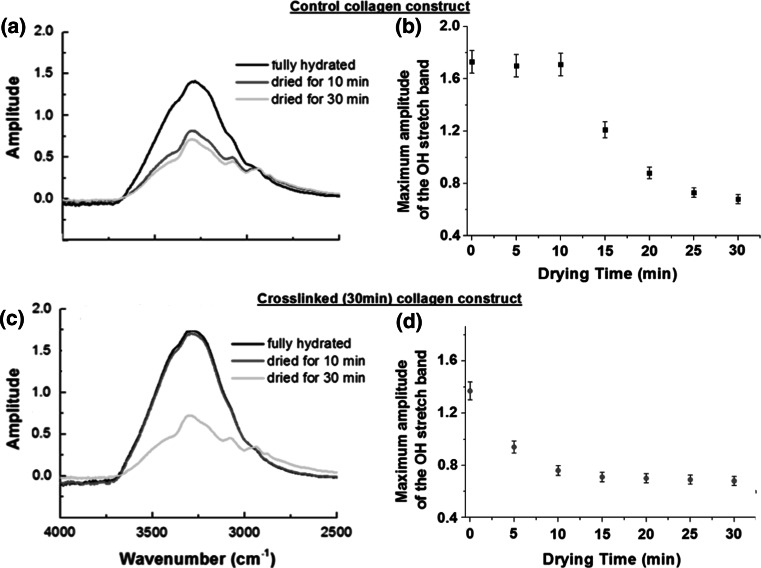
Infrared spectra of the OH-stretch band from **a** control collagen construct and **c** 30 min crosslinked collagen construct recorded after drying for 0 min (*black*), 10 min (*red*) and 30 min (*green*) on the ATR window of the FTIR spectrometer. The control collagen construct dehydrates faster (see **b**) when compared to the cross-linked construct (see **d**) which exhibits a delay up to 10 min before starting to dehydrate as well. The maxima of the OH-stretch band were taken for each spectrum at 3,320 cm^−1^ (Color figure online)

By time-lapse recording every 5 min the amplitude of the OH stretch as collagen samples were left to dry out on the ATR crystal (diamond window) under ambient conditions, it became possible to study the affinity for unbound water for respective collagen constructs. Figure 4 present the OH stretch band recorded on the control collagen construct (a) and the 30 min crosslinked collagen construct (c) immediately after being mounted on the ATR crystal and after respectively 10 and 30 min drying time. In Fig. 4b, d, the maximum amplitude of the OH-stretch (measured 3,320 cm^−1^) is plotted a function of the drying time over the 30 min period. In the case of the control collagen constructs, the amplitude of the water band starts decreasing straight away suggesting that the sample starts drying immediately. However, in the case of the riboflavin crosslinked collagen construct, the amplitude of the OH-stretch remained unchanged for up to 10 min, before it started decreasing similarly to the control construct. This behaviour of the crosslinked collagen construct suggests that interstitial water may have a greater affinity for the crosslinked collagen construct than for the control construct. The process of photochemical crosslinking using riboflavin generates two by-products which lead to the presence of two mechanisms available to interact with the collagen structure. In the direct mechanism (type I), the light activated riboflavin interacts directly with the collagen molecule by hydrogen abstraction of amines to stabilise its own C=bonds therefore enabling intermolecular covalent crosslinks to form in the collagen. The reduced form of riboflavin takes part structurally in the crosslink chain between the individual collagen molecules. Such crosslinks are defined as Non-Enzymatic Glycation (NEG)-induced cross-links, known also as Advanced Glycation End-products (AGEs) [47–49]. Riboflavin is a hydrophilic compound due to the four –OH groups present in its chemical structure. Thus, the affinity of the reduced riboflavin for water could explain the water retention behaviour of the crosslinked collagen construct observed during the drying experiment. Little is known about the influence of water on the collagen fibril microstructure and reported findings are contradictory. In an atomic force microscopy study [50], Kato et al. found no differences in the banding period between dry and wet fibril states, and thus concluded that the axial assembly of collagen molecules is not affected by the intra-fibrillar water. However, in X-ray diffraction data from bulk tendon [51], Wess and Orgel observed an increase in variability of the molecular packing (change in the molecular direction) upon fibril dehydration, together with an simultaneous change in the D-banding periodicity from 67.2 to 64.7 nm.

### Crosslinks behaving as nanosprings

**Fig. 5:**
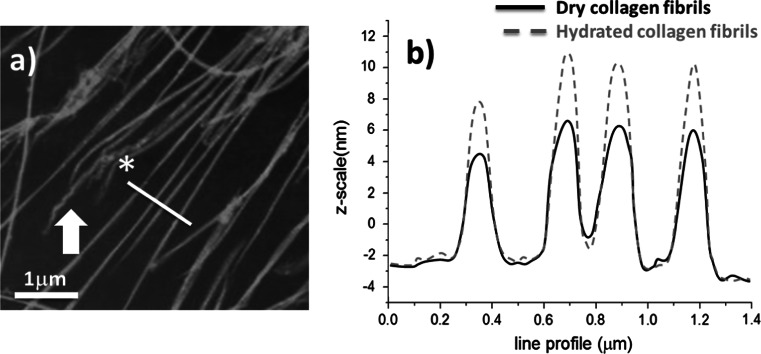
**a** AFM image of collagen fibrils at the edge of a collagen construct (30 min exposed to activation lamp). The *arrow* points towards the end of a fibril showing localised areas of damage (possible unwinding). The *asterisk* highlights the line profile location displayed in **b**. **b** The line profiles of the same hydrated and dehydrated fibrils are plotted showing a significant increase in the fibril cross-sectional area (+68 % for this sample)

Water has a strong influence on collagen constructs but also on individual collagen fibrils. Upon hydration, water leads to radial swelling and softening of fibrils, as previously demonstrated by Robins et al. [52], and modifies its chemical structure through hydrolysis, solvation of free radicals, hydrogen bond stability, and the rate of gelatine formation. Water also influences the fibril structure [51] and the intermolecular crosslink configuration [53]. To investigate the effect of water on individual fibrils from the crosslinked collagen constructs, atomic force microscopy imaging was performed on the same sample successively in dry and hydrated conditions with the aim to measure the profile of individual collagen fibrils. Figure 5a is an AFM image of individual collagen fibrils (hydrated condition) obtained from a crosslinked construct (15 min exposure). Although the fibrils were crosslinked, it is possible to observe local damage at the end of some fibrils (arrow in Fig. 5a), such as a localised unwinding, which is similar to previous published work [54]. As individual fibrils are imaged on the edge of the collagen constructs, such damage is expected as this is the region where the construct was cut. Cross-sectional measurements were not performed in regions of visible damage to fibrils. Figure 5b presents the cross-sectional data of the same series of fibrils in both the dry and hydrated state. From this, an increase in fibril diameter upon hydration can be observed. The fibrillar cross-sectional area was measured using an integration method. Fibrils originating from control collagen constructs had an overall increase in fibrillar cross-sectional area of (42 ± 7) % N = 40 as presented in Table 1. Similar measurements performed on fibrils obtained from two separate crosslinked constructs with exposure times of 2.5 and 30 min produced an overall increase in fibrillar cross-sectional area of (45 ± 8) % N = 36 and (78 ± 12) % N = 45 respectively. For these results, one can deduce that fibril swelling is increasing as a function of activation lamp exposure time, and therefore as a function of the number of crosslinks formed. In the case of the collagen constructs with 30 min exposure, the fibrils almost double in diameter. In the control sample, a swelling is also recorded but the overall increase is still less than the one observed in the riboflavin crosslinked fibrils. A similar result was observe by Wollensak et al. [55], whilst crosslinking rabbit cornea using riboflavin/UVA. To explain the variation in the fibril diameter increase, the presence or absence of riboflavin crosslinks in the individual collagen fibrils has to be taken into account. Considering that the riboflavin crosslinked construct exhibits a greater affinity for water and that individual collagen fibrils can also increase their diameter more when compared to control fibrils (non-crosslinked), the role and the function of the crosslinks must be considered when discussing the collagen ultrastructure. Crosslinks have always been described as chemical bridges that reinforce collagen fibrils and confer greater mechanical properties to the overall construct. However, in this approach we have demonstrated that the there is a second role to the collagen crosslinks related to the water movement in the collagen ultrastructure. Due the hydrophilic nature of the reduced form of riboflavin, water can be drawn into and released out of the fibrils. This concerns only the interstitial water which is not used to stabilize the triple-helix. It means that the crosslinked chains created by the reduced form of riboflavin could effectively act as a hydrophilic nano-spring in between collagen molecules, which would permit the fibril to swell in the presence of water.

**Table 1:** Summary of the mean percentage increase of fibrils cross-sectional area as a function of the light activation exposure time

Crosslinking exposure time	None (control)	2.5 min	30 min
Mean percentage increase in fibrillar diameter upon hydration	42 ± 7	45 ± 8	78 ± 12
Number of fibrils measured	40	36	45

### Impact of covalent nanospring crosslinks on collagen structure and function

Crosslinked chains that have the potential to react and change their conformation depending on the presence or not of water, would have a significant impact on loaded tissue structure and function. Modeling the covalent crosslink formed following the activation of riboflavin as an intermolecular hydrophilic nanospring offers a possible explanation as to how individual collagen fibrils may have a greater affinity for water whilst being able to significantly increase their cross-section area. However, the impact of an individual type of crosslink on the collagen fibril structure and function has not yet been the subject of a systematic study and thus the extrapolation of the mechanical role of the crosslinks remains a hypothesis. The reason for the lack of systematic study on individual crosslinks is probably due to the fact that in vivo, collagen fibrils contain different subsets of crosslinks and thus rationalizing the impact of one type is very difficult. As mentioned before, the type of crosslinks generated by a photochemical crosslinking method belongs to the non-enzymatic glycation (NEG)-induced crosslink family, also known as advanced glycation end-products (AGEs). The significance of these crosslinks is that they occur naturally within the human body and are known to increase with age [56] and occur spontaneously in the presence of high levels of extracellular sugars [57]. However, when the crosslink density increases, as found in elderly individuals, effects can occur such as skin wrinkling, cartilage impairment and brittle bone, as in osteoporosis and diabetes [56, 58]. Thus, it is accepted that any excess of AGEs-related crosslinks may compromise the tissue function and that glycation is a major contributor to the change in mechanical properties associated with ageing for example.

### Future potential of riboflavin collagen crosslinking

In this study, we have demonstrated that a careful control of the degree of crosslinking may offer a new route to increase the mechanical properties of collagen constructs. Plastic compressed collagen was employed by Hu et al. [59] to generate highly functional and mechanically stable dermo-epidermal skin substitutes. In their approach, they dermal fibroblasts were seeded within and co-compressed with hyper-hydrated collagen in order to produce a skin-equivalent. After incubation in submerged conditions for 5 days and 16 days with an air–liquid interface, the compressed collagen construct presented a skin-like morphology. However, the strength property of this construct was not adequate enough for immediate implantation [59]. It is clear from various researches conducted [7], that there is a need for mechanically stronger collagen constructs to be used as tissue engineering scaffolds. As such the work proposed by Mi et al. [38], Wollensak et al. [60] and Caporossi et al. [61] offers direct clinical applications of the process used in our work. In the work presented by Mi et al. [38], riboflavin crosslinkling has been used in the treatment of progressive keratoconus [61, 62]—the most common degenerative dystrophy where the cornea loses mechanical stability. Although the success was limited due to the overall fragility of the scaffold, their work confirmed the validity of this novel approach to rapidly enhance the mechanical properties of the collagen constructs. The application of this protocol could be in fact translated to any engineered collagen scaffolds, provided that a potential decrease in cell viability would be taken into account as described by Cheema et al. [37]. The reduction in the cell viability comes from the fact that any cells in contact with riboflavin would be affected by singlet oxygen toxicity and undergoes cell death after cross-linking. In 2007, Ibusuki et al. [19] indicated that chondrocyte viability after photochemical cross-linking (in 0.25 mM riboflavin) was almost halved by increasing the exposure time from 30 to 600 s, proving that cell viability was illumination time dependent. More recently, Sando et al. [63] used another photochemical agent (ruthenium metal complex) to cross-link a fibrinogen and gelatine scaffold seeded with mouse myoblasts. They observed initially a good in cell viability of (80–90 %), which subsequently dropped to 10 % after 24 h. Cell numbers were, however, recovered after allowing time for proliferation of the surviving cells (1–2 weeks of incubation). These examples of research using photochemical agents to crosslink collagen based scaffolds are all in agreement regarding cell viability. The chemical photo-initiation of the crosslinking agent, riboflavin in our case, has a detrimental effect to the cells present within the scaffold. One hypothesis is that the surviving cells embedded within the collagen matrix would likely be ready for proliferation to ‘replenish’ the cells lost during the cross-linking process. It is however clear that the activation time of the riboflavin is critical to minimise cell death and improve the mechanical properties of the scaffold. One possibility for the correct application of such a protocol might be to increase the seeding density prior to the crosslinking to ensure a sufficient number of cells survive and start proliferating or simply to seed the scaffold only once it has been cross-linked.

In terms of crosslinking method, the range of applications of this crosslinking approach will be defined by its relative simplicity and speed when compared to other methods relying on cellular activity, aldehyde or glycation processes. However, it is also clear that riboflavin crosslinking confers properties to collagen constructs that are not yet comparable to those of native tissue and thus should be considered as method of crosslinking to be used complementarily. Yet using this approach to engineer cross-linked collagen phantoms of disease, such as fibrosis for example, may hold some promises towards the elucidation of the biophysical properties of cross-linked collagen at the fibrillar level for example.

## Conclusion

The use of collagen constructs for TE applications holds tremendous promise as it circumvents the complications associated with synthetic polymers. Cellular organisation and the corresponding characteristics imparted to the tissue are highly dependent on the complex structure of the ECM. Current macro- and micro-fabricated constructs fail to recreate the natural nanostructures that our cells interact with. The fibrils in collagen constructs, both morphologically and chemically, mimic the effects of natural ECM, giving excellent biocompatibility for clinical applications. However, it is important that the construct has the same mechanical properties as the target tissue in order to tolerate natural stresses and restore function with a desirable cosmetic outcome. Untreated collagen is mechanically weak and has a tendency to deform given its weak lattice structure when compared to naturally formed collagen. Using a photochemical crosslinking protocol that is fast, efficient, affordable and refined by the addition of complementary techniques such as plastic compression offers new possibilities for the whole field of tissue engineering. Optimized engineering protocols would help develop clinical applications such as soft tissue augmentation—using photochemically reinforced constructs that are non-cytotoxic and otherwise biocompatible; restoration of allografts that deteriorate on sterilization—ionizing radiation is used in the removal of resistant pathogens; and for other bioprostheses including nerve conduits, cartilage, heart valves and dural substitutes [19, 36].
